# A Segmentation-Guided Deep Learning Framework for Leaf Counting

**DOI:** 10.3389/fpls.2022.844522

**Published:** 2022-05-19

**Authors:** Xijian Fan, Rui Zhou, Tardi Tjahjadi, Sruti Das Choudhury, Qiaolin Ye

**Affiliations:** ^1^College of Information Science and Technology, Nanjing Forestry University, Nanjing, China; ^2^School of Engineering, University of Warwick, Coventry, United Kingdom; ^3^Department of Biological Systems Engineering, University of Nebraska-Lincoln, Lincoln, NE, United States

**Keywords:** plant phenotyping, segmentation, deep CNN architecture, leaf counting, multiple traits

## Abstract

Deep learning-based methods have recently provided a means to rapidly and effectively extract various plant traits due to their powerful ability to depict a plant image across a variety of species and growth conditions. In this study, we focus on dealing with two fundamental tasks in plant phenotyping, i.e., plant segmentation and leaf counting, and propose a two-steam deep learning framework for segmenting plants and counting leaves with various size and shape from two-dimensional plant images. In the first stream, a multi-scale segmentation model using spatial pyramid is developed to extract leaves with different size and shape, where the fine-grained details of leaves are captured using deep feature extractor. In the second stream, a regression counting model is proposed to estimate the number of leaves without any pre-detection, where an auxiliary binary mask from segmentation stream is introduced to enhance the counting performance by effectively alleviating the influence of complex background. Extensive pot experiments are conducted CVPPP 2017 Leaf Counting Challenge dataset, which contains images of Arabidopsis and tobacco plants. The experimental results demonstrate that the proposed framework achieves a promising performance both in plant segmentation and leaf counting, providing a reference for the automatic analysis of plant phenotypes.

## Introduction

Plant phenotype is a set of observable traits of a plant, which is heavily influenced by the interaction between plant gene expression and environmental factor (Siebner et al., 2009). The accurate and efficient monitoring of phenotypes is essential for plant cultivation, which is a prerequisite for intelligent production and planting, and information/data management. The traditional monitoring of plant phenotype mainly requires manual observation and measurement to analyse the appearance of plants in terms of their shape, texture, colour, and other characteristic morphological phenotypes (Montero et al., 2000; Minervini et al., 2015). Such an approach is labour intensive, which is time-consuming and prone to error due to the reliance on subjective perception (Yang et al., 2020). Image-based plant phenotyping allows non-invasive and distant observation, reducing the effects of manual interference and vastly increasing the scale and throughput of plant phenotyping activities. However, it still requires a robust algorithm to automatically process the input image to provide accurate and reliable phenotypic estimation (Scharr et al., 2016). In addition, such an algorithm should be able to estimate a wide diversity of phenotypes, which allows for a range of different scientific applications. The current trend of image-based plant phenotyping attempts to combine image processing (e.g., noise removal and image enhancement), feature extraction and machine learning to obtain effective and efficient estimation (Tsaftaris et al., 2016). In recent years, deep learning-based methods have made remarkable progress in the field of computer vision such as semantic segmentation, classification, and object detection (Lecun et al., 2015). They integrate feature extraction and classification using a single convolutional neural network (CNN) based framework, which is trained in an end-to-end fashion. Due to their powerful ability to capture meaningful feature representation, deep learning-based methods are drawing more attention in the plant research community (Dhaka et al., 2021; Kundu et al., 2021) and have also been applied to deal with different tasks in plant phenotyping (Choudhury et al., 2019).

Plant segmentation and leaf counting are two fundamental tasks of plant phenotyping as they are relevant to the developmental stage of a plant, and are considered essential means of providing vital indicators for the evaluation of plant growth (e.g., growth regulation and flowering time), yield potential, and plant health. Moreover, they help farmers and horticulturists to make better decision regarding cultivation strategic and timely horticulture adjustments. Plant segmentation aims to extract the plant area, shape, and size from a visual perspective by segmenting an entire plant from the scene background in an image. Such a task closely relates to the semantic/instance segmentation problems, and some researchers have addressed this task using instance/semantic segmentation (Romera-Paredes and Torr, 2016; Ren and Zemel, 2017; Ward et al., 2018; Zhu et al., 2018), achieving promising performance. Leaf counting aims to estimate the precise number of leaves of a plant. There are two mainstream ways to infer the leaf count or leaf number: (1) estimating the leaf number as a sub-product of leaf segmentation or detection (Girshick, 2015; Kong et al., 2020; Kumar and Domnic, 2020; Lin and Guo, 2020; Lu and Cao, 2020; Tassis et al., 2021); and (2) directly regarding the task as a holistic regression problem (Dobrescu et al., 2017; Giuffrida et al., 2018; Itzhaky et al., 2018; Ubbens et al., 2018; Mishra et al., 2021). The methods have successfully addressed the tasks of leaf segmentation and counting using machine learning and especially deep learning methods, which uncover the intrinsic information from plant images, even when they contain complex structure. However, they merely focus on a single task, i.e., learn one plant trait at a time. Thus, they might ignore the facts that plant phenotype traits tend to be associated with each other and lack the insight to the potential relationship between different traits (Gomes and Zheng, 2020). For instance, the leaf number is associated with the leaf area, age, and genotype. We believe that incorporating multiple traits in the deep CNN architecture could be beneficial for learning more reliable and discriminative information than using only one trait. Dobrescu et al. (2020) presented a multi-task framework for leaf count, projected leaf area, and genotyping, where they compute three plant traits at the same time by using the share representation layers. However, they did not address the tasks of plant segmentation that is more challenging due to the requirement of classifying all the leaves (foreground) pixel by pixel.

Convolutional neural network based methods have been applied to plant and leaf segmentation in plant phenotyping. Aich and Stavness (2017) used a CNN based deconvolutional network for plant (foreground) and leaf segmentation. Kuznichov et al. (2019) utilised data augmentation technology to maintain the geometric structure and physical appearance of plant in images to improve the leaf segmentation. Bell and Dee (2019) employed a relatively shallow CNN model to classify image edges extracted using Canny edge detector, which distinguished the occluding pairs of leaves. Ren and Zemel (2017) adopted recurrent neural network (RNN) to generate a single segmented template for each leaf and combined convolutional long short-term memory (LSTM) network using spatial inhibition modules. They then used dynamical non-maximal suppression to leverage the previously segmented instances to enhance the segmentation. Although achieving promising results, these methods use the shallow CNN model, which is inadequate to capture the meaningful information of the diversity of plant images. Moreover, all methods concentrate on addressing the single task, i.e., leaf/plant segmentation in an independent pipeline.

Image segmentation using deep learning has gained a significant advance, and a few benchmark methods have been proposed. Fully convolutional networks (FCN) (Long et al., 2015) and U-Net (Ronneberger et al., 2015) are two representative models that are based on the encoder-decoder network architecture. Both of them share a similar idea, i.e., using skip connection, that shows the capability to capture the fine-grained characteristics of the target images. FCN summed the up-sampled feature maps with feature maps skipped from the encoder, while U-Net modified the way of feature concatenation by adding convolutions and non-linearities during each up-sampling step. Another mainstream work is using spatial pyramid pooling ideas. PSPNet employed a pyramid parsing operation that captures global context information by region feature aggregation (Zhao et al., 2017). DeepLab (Chen et al., 2017) introduced the atrous convolution with up-sampling filter for feature extraction, and extended it using spatial pyramid pooling to encode the multi-scale contextual semantics. However, the various scale pooling operations tend to lose local spatial details and will fail to maintain leaf target with high density if a small input size is adopted. The Mask Region Convolutional Neural Network (Mask-RCNN), proposed by He et al. (2017), extended the region proposal network by integrating a branch to predict segmentation mask on each ROI. Mask RCNN can segment the object with pixel-wise mask from a complicated background, which is suitable for the leaf segmentation. Thus, we developed our network model based on the backbone architecture in Mask-RCNN and simply replaced the plain skip connection with a nested dense skip pathway to enhance the ability to extract more fine-grained features in leaf images.

Leaf counting is also an important task in plant phenotyping, since leaf count is considered as an indicator for yield potential and plant health (Rahnemoonfar and Sheppard, 2017). From the perspective of computer vision, leaf counting can be addressed along two different lines: (1) Regarding leaf counting as the sub-product of leaf segmentation or detection, leading to the leaf number following the segmentation module; and (2) Directly learning an image-to-count model to estimate the leaf number using training samples.

### Direct Count

Leaf counting is regarded as a holistic regression task, in which a counting model estimates the leaf number for a given plant image. In this way, the machine learning based regression model solely needs the annotation of leaf number, which is an easier way to obtain compared with the pixel-wise annotations using segmentation. Dobrescu et al. (2017) presented a counting framework employing the ResNet50 backbone (He et al., 2016), in which the learning of leaf counting is performed by gathering samples from multiple sources. Itzhaky et al. (2018) proposed to estimate the leaf number using multi-scale representations and fuse them to make the final predictions. Ubbens et al. (2018) presented an open-source platform which aims to introduce a more generalised system for plant breeders, which can be used to count leaves across different datasets, as well as to assist other tasks e.g., projected leaf area and genotype classification. da Silva and Goncalves (2019) constructed a CNN based regression model to learn from images, where the skip connections of Resent50 (He et al., 2016) are considered efficient for leaf counting. Direct count could be a natural and easy selection as it is not necessary to annotate the image when training.

### Counting via Detection or Segmentation

This approach regards the leaf counting problem as a sub-product of detection or segmentation, where the exact locations and number of the leaves are also obtained after detection or segmentation. Romera-Paredes and Torr (2016) proposed to learn an end-to-end segmentation model using RNN, that segments each leaf sequentially and then estimate the number of segmented leaves. Aich and Stavness (2017) used a CNN based deconvolutional network for leaf segmentation and a convolutional network for leaf counting. Kumar and Domnic (2019) developed a counting model with the combination of CNN and traditional methods, where graph-based method is used for U-Net segmentation and CNN-based is then used for leaf counting via a fine-tuned AlexNet. Ren and Zemel (2017), propose a neural network using which visual attention operation to jointly learn the instance segmentation and counting model, where sequential attention using LSTM cell is created by using temporal chain to output one instance at a time. However, such a segmentation or detection-based method has one limitation for counting. That is, only successfully segmented leaves are counted, and imperfect detection will result in reduced accuracy in counting. Unlike the aforementioned methods, we employ the segmented binary image to guide the learning of leaf counting, i.e., not counting directly from the segmented image, thus avoiding the effect of inaccurate detection or segmentation on the counting task.

In this study, we present in this article a two-stream framework, one stream for plant segmentation and the other stream for leaf counting based on regression. The resultant mask from segmentation stream is leveraged to guide the learning of leaf counting, which help to alleviate the inference of complex background. In order to obtain more semantic and meaningful feature representation of plant images, we employ the deep CNN as the model backbones of both two streams. By using the CNN paradigm, the two-stream model is robust and generalizes well regardless of the plant species and the quality of the acquired image data. This is achieved by one stream task supervising the training of the other stream task *via* sharing certain knowledge. To this end, we employ the segmented binary mask from the plant segmentation stream as an auxiliary cue to optimise the training process of the leaf counting stream. Introducing the binary mask to supervise the learning of leaf counting is based on two issues that exclusively exist in plant leaf counting: (1) some leaves might be partially occluded by other leaves, or are incomplete and fragmentary on their own, making them difficult to detect; and (2) the leaves sometimes contain complex background, increasing the challenge in leaf counting. These two issues led to incorrect or missing count where the meaningful and useful information of leaf is hard to maintain during the leaf counting. The binary mask effectively deals with these two issues by precisely locating all individual leaves while alleviating the effect of complex background. In addition, the binary mask of image samples brings more diversity of the input images by increasing the number of samples, which could be regarded as an implicit data augmentation.

Specifically, in our proposed framework, a two-stream deep neural network model segments the leaves and counts the number of leaves, where the segmented binary mask is employed as an auxiliary cue to supervise the learning of leaf counting. In the stream for segmentation, a multi-scale-based segmentation network is proposed to extract fine-grained characteristics of leaves. In the stream for leaf counting, we propose to learn a regression model based on the fine-tuned CNN model. During the learning of leaf counting, the segmented mask is utilized to highlight the target leaf region (foreground) of interest (ROI) from the entire image by removing the disturbance of complex background (i.e., non-leaf area, thus facilitating the counting process.

The contributions of this study are summarized as follows:

1. We propose to explore fine-grained characteristics, i.e., high inter-class similarity and low intra-class variations, widely existing in high throughput plant phenotyping that cause the failure in localizing the leaves within a small area during segmentation. To address this issue, we introduce a multi-scale U-Net segmentation model which compensates the upper-lower semantics difference by concatenating features in various scales. This model is learned in an end-to-end fashion, allowing for efficient segmentation of the leaves with different areas.
2. We propose a two-stream network based on deep CNN architecture to complete the leaf counting together with plant segmentation, in which the model outputs the segmentation results and directly estimates the leaf number.
3. We enhance the leaf counting by introducing the auxiliary binary information. The binary mask is utilised to supervise the leaf counting, which increases the contrast between the leaf target from background interference, and significantly aids the convergence of the counting regression model.

The remainder of the article is presented as follows: we review related work in Section “Introduction,” present our method in Section “Proposed Method,” provide the experimental results in Section “Experiments” and discuss the conclusions and further work in Section “Conclusion.”

## Proposed Method

**FIGURE 1:**
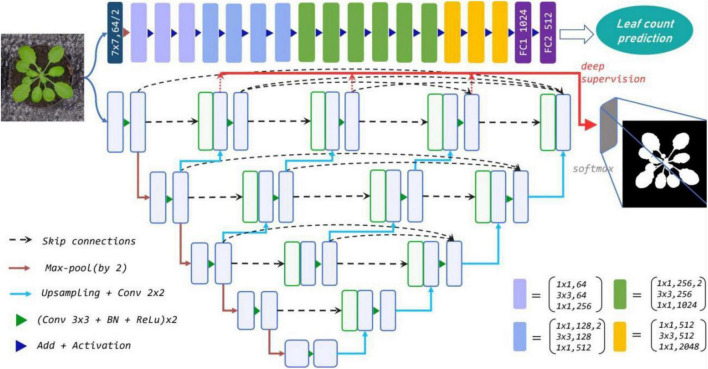
The proposed parallel two-stream network combines leaf counting and segmentation tasks. Top row: the modified Resnet50 regression model for leaf counting with 16 residual blocks. Remaining rows: U-Net++ for segmentation *via* multi-use of the features from different semantic levels (layers). Each blue box corresponds to a multi-channel feature map, and the green boxes represent copied feature maps. The arrows denote various operations.

We present a parallel two-stream network for determining leaf count and undertake segmentation simultaneously for the rosette-shaped plants as shown in Figure 1. The stream for segmentation adopts the nested U-Net (U-Net++) architecture (Zhou et al., 2018) as backbone to extract the target leaf region from the entire image using a binary mask. The stream for leaf counting learns the CNN based regression model which is customized by modifying its last layer to directly estimate the number of leaves where the segmented mask and original colour images with the leaf number label are mixed as input of the regression model. The streams for plant segmentation and count are designed separately first. The segmented binary mask denoting the area of the leaf is used as a complementary cue to supervise the learning of the count regression stream. This is because the two key traits of the two streams, i.e., the area and leaf number are often related to each other. Incorporating the leaf area into the estimation of leaf number during the learning of deep neural network aids not only to learn more meaningful and essential information, but also alleviates the influence of complex background.

### Plant Segmentation Module

The segmentation module aims to extract the whole leaf area from the background. In order to enhance the robustness and accuracy of extraction, it is a necessity for the module to be in capacity to depict the characteristics existing in a plant image, i.e., fine-grained and variation in shape and size. To this end, we consider the nested U-Net as our backbone network for the segmentation. The nested U-Net model is proposed based on the U-Net that was originally proposed to meet the requirement on accurately segmenting medical images. Compared with the original U-Net model proposed by Ronneberger et al. (2015), the nested U-Net architecture replaces the plain skip connection with nested and dense skip connections, which can capture fine-grained information of the object in an image. Moreover, due to the up-sampling scheme, the U-Net model could locate leaves with different size and shape by using feature maps with different scales. By dealing with the characteristics in leaves, the nested U-Net is thus suitable for plant segmentation. Another problem needs to be addressed during training, namely the ROIs of plant segmentation comprise a relatively small segment of the entire image. Thus, negative samples (i.e., background pixels) are much larger than positive samples (i.e., leaf pixels), which resulted in an unbalanced binary classification problem. To address the problem, we integrate the binary cross-entropy (BCE) loss with dice loss together, and jointly guide the learning process of the segmentation. Generally, the nested U-Net consists of three main modules: encoding, decoding, and cross-layers dense concatenation. The feature maps in the same size are defined to be of the same layer, denoting the layers as L1–L5 from top to bottom. Each node represents a feature extraction module consisting of two 3 × 3 convolutional layers, followed by a rectified linear unit (ReLU) and a 2 × 2 max pooling that use stride 2 for down-sampling.

The output features from encoder are fused with the next encoder layer *via* up-sampling features across layers from top to bottom. The fusion outputs are concatenated with the corresponding up-sampled features of the next layer, and the process is iterated until there is no corresponding module in the next layer. The integrated feature maps are defined as


(1) $$x^{i,j}=\left\{ \begin{array}{cc} ℋ\,\left(x^{i-1,j}\right) & j=0\\ ℋ\,\left(\left[{\left[x^{i,k}\right]}_{k=0}^{j-1},𝒰\,\left(x^{i+1,j-1}\right)\right]\right) & j>0 \end{array}\right.$$

where ℋ(⋅) denotes a convolution operation followed by an activation function, 𝒰(⋅) denotes an up-sampling layer, and [] denotes the concatenation layer. Nodes at level *j* = 0 only receive input from the previous encoder layer; nodes at level *j* = 1 receive the encoder and sub-network input from two consecutive levels; and nodes *j* > 1 receive *j* + 1 inputs of which j inputs are the outputs of the previous j nodes in the same skip pathway and the last input is the up-sampled output from the lower skip pathway.

The dense skip connections between layers in the same dimension pass the output of the current module to all subsequent modules and fuse it with other input features. Thus, the overall U-Net++ feature fusion structure is in the form of an inverted pyramid, where the intermediate layer contains more accurate localisation information, while the in-depth layer captures pixel-level category information.

As a typical binary classification task, the core objective is to segment the plant image into a binary image by labelling the foreground and background pixels as 1 and 0, respectively. To overcome the class imbalance problem, BCE loss and Dice loss are combined to form the objective function to optimize the imbalance between the foreground and background pixels through back-propagation. Dice coefficient is a measure of the pixel degree of an ensemble, and the original expression takes the form of


(2) $$d=\frac{2\,\left|X\cap Y\right|}{\left|X\right|+\left|Y\right|}$$

where *X* and *Y* are sets, and *s* ∈ [0, 1], and the size of s reflects the similarity between the sets *X* and *Y*.

The binary cross-entropy and dice coefficient are combined to form the final loss function, which is defined as


(3) $$ℒ\,\left(Y_{g\,t},Y_{p\,r\,e\,d}\right)=-\frac{1}{N}\,\sum_{b=1}^{N}\left(\frac{1}{2}\cdot Y_{g\,t}^{b}\cdot \text{log}\,Y_{p\,r\,e\,d}^{b}+\frac{2\cdot Y_{g\,t}^{b}\cdot Y_{p\,r\,e\,d}^{b}}{Y_{g\,t}^{b}+Y_{p\,r\,e\,d}^{b}}\right)$$

where *Y*^*b*^_*gt*_ and *Y*^*b*^_*pred*_ denote the predict map and ground truth map of *b*-th image, respectively, and *N* denotes the batch size.

The objective function takes the form of a logarithmic logic function as a replacement for the complex softmax multi-class prediction function. Forward propagation infers the prediction results and compares them with the true value annotations to generate cross-entropy loss. Backward propagation updates the model weight parameters. In this way, the task of plant segmentation is transformed into a binary classification problem that is suitable for plant segmentation. The re-designed skip pathways take effect on the output of the fused features and simplify the optimisation on the shallow, middle, and profound output results for varying degrees, *via* tuning the overall parameter of the network.

### Learning Count Model With Segmentation

During leaf counting, the estimated number of leaves tends to exceed its ground truth. This is because the lower part of a leaf might be occluded by other leaves, or the leaves are incomplete and fragmentary on their own, which would be ignored by the counting model. To address this problem, we introduced the auxiliary cue, i.e., the segmented mask to guide the learning of the counting model. Also, it is widely acknowledged the counting model could fail due to the lacking of available samples belonging to certain class in the training dataset. The labelling for leaf counting is also time-consuming. Such data scarcity is often met in the data-driven methods such as deep learning. Thus, we augmented the samples by combining the segmented mask and the original images, which enhance the model to effectively capture the occluded leaves and the hardly detected leaves in plant image under the assistance of segmented binary mask.

Inspired by the work of He et al. (2016), we employed Resnet50 network as our backbone architecture due to its superb performance in image recognition. For our regression task, we modified the Resnet50 network by replacing the last layer with a fully connected layer with one-dimension output, which acts as a regression model for leaf counting. The modified network uses the combined samples from the segmentation mask and the original images as input, and applies convolution with a 7 × 7 filter followed by a series of convolutions, ending with fully connected layers to determine the number of plant predictions. Residual learning is also used to overcome the inefficient learning and the possibility of over-fitting due to deep network, where the skip connections resolve the degradation problem by taking the output of the previous layers as the input of the latter. For instance, when an input is x and the learned features are denoted as *H(x)*, then the residual learning features is *F(x) = H(x) - x*. The stacked-layer learns new features on top of the input features, and a residual unit is given by


(4) $$y_{l}=h\,\left(x_{l}\right)+F\,\left(x_{l},W_{l}\right),x_{l+1}=f\,\left(y_{l}\right)$$

where *x*_*l*_ and *x*_*l + 1*_, respectively, represent the input and output of the l th residual unit, and each residual unit contains multiple layers of structure. *F* represents the learned residual block, *h(x_*l*_) = x_*l*_* is the constant mapping, *f* is the ReLU activation function. Thus, the learned features from shallow *l* to deep *L* are


(5) $$x_{L}=x_{l}+\sum_{i=l}^{L-1}F\,\left(x_{i},W_{i}\right)$$

A chain rule is used to aid the reverse process of gradients, i.e.,


(6) $$\frac{\partial \mathrm{loss}}{\partial x_{l}}=\frac{\partial \text{loss}}{\partial x_{L}}\cdot \frac{\partial x_{L}}{\partial x_{l}}=\frac{\partial \text{loss}}{\partial x_{L}}\cdot \left(1+\frac{\partial }{x_{L}}\,\sum_{i=l}^{L-1}F\,\left(x_{i},W_{i}\right)\right)$$

where $\frac{\partial \text{loss}}{\partial x_{L}}$ denotes the gradient of the loss function reaching *L*, the value 1 in the parentheses indicates that the shortcut connection mechanism propagates the gradient without loss, while other residual gradient passes through a layer with weights indirectly. In this context, 1 is selected to make the residual gradient easier to learn and thus avoid the gradient vanishing.

To better train the regression model, we employed mean squared error (MSE) as the loss function. Given an image *i* and the ground truth leaf count *y*^*i*^_*gt,c*_, the loss function *L*_*c*_ is determined by


(7) $$L_{c}=\frac{1}{m}\,\sum_{i=1}^{m}{\left(y_{p\,r\,e\,d,c}^{i}-y_{g\,t,c}^{i}\right)}^{2}$$

where *m* is the image number and *y*^*i*^_*pred,c*_ denotes the predicted leaf count.

With respect to our regression task, the last fully-connected layer with 1,000 neurons initially used for classification is replaced by a layer with a single neuron, which allows for the output estimation of leaf number. The neuron is to regress the correct leaf numbers given the input images. To obtain the rich prior knowledge, the regression network is pre-trained on ImageNet for parameter initialization, and then fine-tuned on the used datasets. Our regression model is shown in the top row of Figure 1. Note that the combination of segmentation and RGB images extends the input channel from 3 to 4. By extending the channel, an additional binary channel is added to the leaf count regression model to convey pure semantic information of leaf and suppress bias from features in the background of the training images, e.g., the soil, moss, pot, etc., that differ between datasets. At the same time, the RGB channels enable the network to retain the rich local texture and context information that the binary mask fails to capture, thus enhancing the robustness of our model. In addition, our regression model does not require any bounding box or centre point annotation, which can be efficiently applied to deal with more complex scenes.

U-Net remains the preferred choice for the maintenance of fine edge binary segmentation. The design of skip connections greatly enriches the information received by the decoder, and *via* specially trained end-to-end, U-Net performs high-precision segmentation for small training samples. When applied in leaf segmentation, the architecture extracts the edge details, size, and shape diversity in the low-level information and uncovers the discriminative high-level information of the target leaf. This advantage reduces the overall size of the dataset required for training. Furthermore, due to the effective reuse of extracted features and an ability to capture the targets, the architecture achieves an implicit data argumentation and speeds up the convergence for the binary tasks during training.

However, since the leaf dataset (with sub-datasets A1–A4) varies in the degree of occlusion, leaf numbers and leaf size, we only combined the same-scale information not previously countered. Designing U-net with different depth for each layer may be an idea but such an approach has not been widely applied. To address this, we adopt U-Net++ (remaining rows of Figure 1) as the feature extractor for segmentation, which extends U-Net with denser cross-layer concatenation and shortens the semantic gap between the encoder and decoder by fusing spatial information from shallow to deep cross layers. The architecture makes full use of contextual features and semantic information from the same dimension, and it captures the detailed features of the target. Moreover, using the pruning scheme basing on the module which receives the best estimation during training, the network is adjustable and customisable. For instance, it is customised to the most suitable size and saves unnecessary storage space. This is equivalent to the maintenance of any useful feature we acquired and the distinctive design for each dataset in one end-to-end network.

## Experiments

We thoroughly assess the effectiveness of our proposed framework on the widely used plant phenotyping dataset including its four sub-datasets (see Section “Dataset and Data Pre-processing”). We conducted extensive experiments on both plant segmentation and leaf counting, and compared the performance of our method with the state-of-the-art methods for validation. We explored three segmentation architectures using three different backbone networks, i.e., MobileNet, ResNet, and VGGNet on the four sub-datasets, and compared our method with the state-of-the-art leaf segmentation methods. We also performed the experiments to demonstrate the effectiveness of the proposed leaf counting method, comparing it with the state-of-the-art leaf counting methods.

### Dataset and Data Pre-processing

The dataset used in our experiments belongs to the Leaf Segmentation and Counting Challenge (LCC and LSC) held as part of the Computer Vision Problems in Plant Phenotyping (CVPPP 2017) workshop (Giuffrida et al., 2015). The dataset is divided into training set and testing set, which consists of 810 and 275 top-down view RGB images of either Tobacco or Arabidopsis plants, respectively. Both training and testing images are grouped into four folders, i.e., four sub-datasets which vary from the species and means of collection such as imaging setups and labs. The training sets include 128, 31, 27, 624 images and the testing sets contain 33, 9, 65, 168 images for A1, A2, A3, and A4 respectively. The sub-datasets A1 and A2 include Arabidopsis images collected from growth chamber experiments with different field of views covering many plants and then cropped to a single plant image with the size of approximately 500 × 500 pixels. Sub-dataset A3 contains tobacco images at 2,000 × 2,500 pixels with the field of view chosen to encompass a single plant. Sub-dataset A4 is a subset of another public Arabidopsis dataset. The dataset provides the corresponding annotations in binary segmentation with 1 and 0, respectively, denoting plant and background pixels. All the folders contain the ground truth binary mask used for whole plant segmentation (i.e., semantic segmentation). For the experiment of plant segmentation, we follow the training strategy from Aich and Stavness (2017), and also use the combination of all sub-datasets (referred as to *All*) for training to achieve more robust model.

In our work, we addressed two problems caused by a dataset as follows: (1) Deep learning based methods require a huge amount of training samples while the availability of the dataset of plant leaf with annotations is limited, causing data scarcity; and (2) Small and overlapping leaf instances brought a challenge for plant segmentation and leaf counting. Data augmentation is a widely used technique in deep learning to increase the number of samples and provide more diversity to the deep neural networks. In this context, we also employed data augmentation to address the above two problems.

**FIGURE 2:**
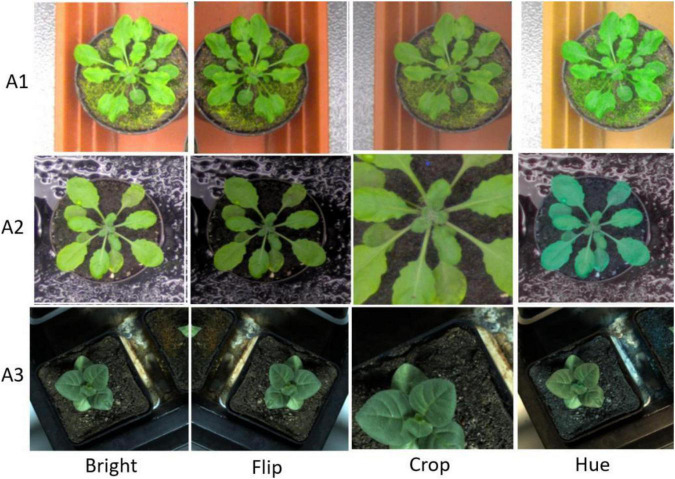
Augmentation samples for training the segmentation network to avoid the risk of over-fitting.

Moreover, we first reshaped the size of training images to 480 × 480 pixels and normalized. Following the resize operation, we conducted the following scheme for data augmentation: (1) Random-Rotate with an interval of 90 to increase the network invariance to slight angular changes; (2) Flip: horizontal, vertical, and horizontal+ vertical; (3) Resize the images to increase the network invariance to different image resolutions; (4) Gamma transform to extend the data by changing the image greyscale; (5) Random-Brightness: the clarity of object depends on scene lighting and camera sensitivity, thus random changing the image brightness improves the illumination invariance of the network; (6) Random change in the contrast range to increase the network invariance to shadows and improve the network performance in low light conditions; (7) Hue Saturation Brightness (HSV): changes in colour channels, degree of lightness or darkness of a colour; and (8) Normalise a characteristic linear transformation which scales a specific range of data values retaining the original data distribution. Selected augmentation processes are shown in Figure 2.

### Implementation Details and Evaluation Protocol

All images from the training set are randomly split into two sets for training and validation with the split ratio of 0.8 and 0.2, respectively. Images from the testing set are used for evaluating the segmentation performance. We used the validation set to verify the hyper-parameters (see Table 1) during the training of the initial experiments.

**TABLE 1:** Hyper-parameters used for training.

Epochs	100
Batch-size	4
Optimizer	Adam
Learning-rate	1e-3
Weight-decay	1e-4
Factor	0.1

#### Network Parameter Setting

All our experiments are performed on the PyTorch platform with NVIDIA 2080Ti GPU. We used the data augmentation to increase the number of samples as in Section “Dataset and Data Pre-processing.” This module contributes to preventing over-fitting for the relatively small plant datasets and ensure the model produces promising results when segmenting on new data *via* learning multiple variations (Holmberg, 2020). The binary mask is transformed the same way, to maintain the consistency between images and annotations (except for the transformation regarding colours).

We randomly sampled four samples to form a mini-batch with batch size of four to guarantee the convergence of training. Adam is adopted as the optimizer for its fast convergence rate to train the model for a total of 100 epochs, where the results remain stable with no further improvement. The weight decay factor is set to 0.0001 and the learning rate is constantly set as 0.001.

#### Metrics for Segmentation

We employed the intersection of union (IoU) as the evaluation metric, which is widely used in segmentation. IoU is used to determine the spatial overlap between the segmented leaf region and its ground truth, i.e.,


(8) $$\text{IoU}\left(\%\right)=\frac{\left|P_{\text{gt}}\cap P_{\text{pred}}\right|}{\left|P_{\text{gt}}\right|+\left|P_{\text{pred}}\right|}$$

where *P*_*gt*_ and *P*_*pred*_, respectively, denote the ground truth mask and the prediction mask. Due to the problem of class imbalance between positive and negative samples, it is insufficient to use accuracy as evaluation metric. For better evaluation, we introduced two more metrics: Precision and Recall. Precision is used to determine the portion of segmented leaf region pixels that matches with the ground truth, i.e.,


(9) $$\text{Precision}\left(\%\right)=\frac{T\,P}{T\,P+F\,P}\times 100$$

Recall is used to determine the portion of ground-truth pixels present in the segmented leaf region, i.e.,


(10) $$\text{Recall}\left(\%\right)=\frac{T\,P}{T\,P+F\,N}\times 100$$

where True Positive (TP), False Negative (FN), and False Positive (FP) respectively denote the number of leaf region pixels correctly identified, the number of leaf region pixels unidentified, and the number of leaf region pixels falsely identified.

#### Metrics for Count

To evaluate how good a leaf count method is in estimating the correct number of leaves, we employed the regression metrics: Difference in Count (DiC), Absolute Difference in Count (ADiC), and mean squared error (MSE) calculated as follows:


(11) $$\text{DiC}=\frac{1}{m}\,\sum_{i=1}^{m}\left(y_{\mathrm{gt},c}^{\left(i\right)}-y_{\mathrm{pred},c}^{\left(i\right)}\right)$$

(12) $$\text{ADiC}=\frac{1}{m}\,\sum_{i=1}^{m}\left|\left(y_{\mathrm{gt},c}^{\left(i\right)}-y_{\mathrm{pred},c}^{\left(i\right)}\right)\right|$$

(13) $$\text{MSE}=\frac{1}{m}\,\sum_{i=1}^{m}{\left(y_{\mathrm{gt},c}^{\left(i\right)}-y_{\mathrm{pred},c}^{\left(i\right)}\right)}^{2}$$

### Experimental Analysis

#### Segmentation Analysis

**FIGURE 3:**
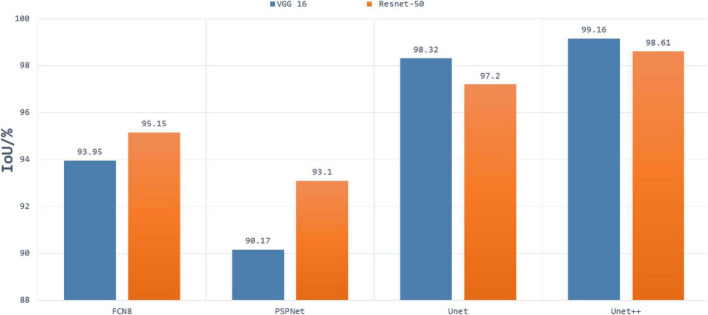
Results of segmentation using Resnet50 and VGG16 as backbone in FCN, PSPnet, U-Net, and U-Net++ architectures.

**FIGURE 4:**
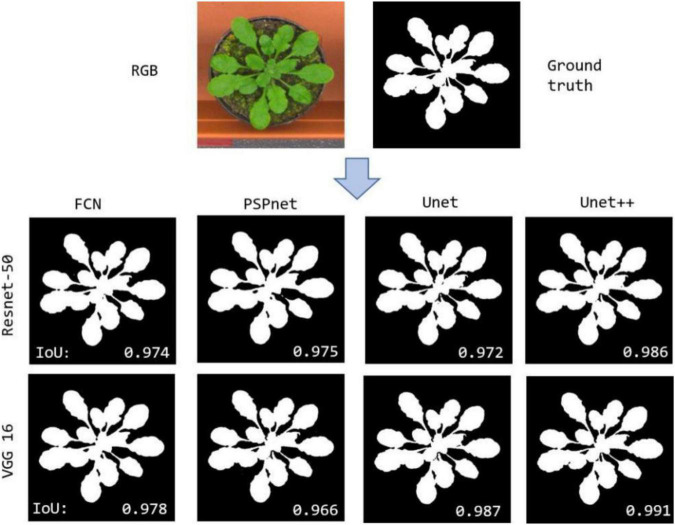
Comparing segmentation results on the same RGB image.

In the first experiment, we evaluated the effectiveness of our segmentation model on plant images by using different segmentation architectures and backbones for comparison. FCN8, PSPNet, U-Net are selected as the basic encoder and decoder architectures, where ResNet and VGG are used as backbones due to its good ability of depicting 2D images. The comparative segmentation performance in terms of IoU on the combination of all sub-datasets are provided in Figure 3. It is evident from Figure 3 that the segmentation results generated by our segmentation model outperforms the other architectures. Among different models, using VGG as backbone performs constantly better than using ResNet as backbone. To evaluate the performance of dealing with a variety of scenes, we evaluated our model on the four individual sub-datasets and the results are shown in Table 2. The U-Net++ performs significantly better than the state-of-the-art segmentation methods. For better illustration, the segmentation results for images in sub- dataset A1 using different models together with ground truth are shown in Figure 4. Although all the three semantic segmentation methods can obtain clear segmentation results on A1, the U-Net++ retains the boundary and detail information. For the relative scarce sub-dataset A3 which only contains 27 tobacco images, the proposed method still shows a stable IoU. For each sub-dataset, the network generates segmentation results that are almost consistent with the corresponding binary template, from both quantitative and qualitative standpoints.

**TABLE 2:** Segmentation results on each sub-dataset and their com- bination using different basic architectures.

IoU (%)	All	A1	A2	A3	A4
FCN	93.95	93.45	89.17	88.51	92.23
PSPNet	90.17	94.34	90.55	91.19	93.83
U-Net	98.32	98.51	97.76	94.72	97.17
U-Net++	99.11	98.29	97.98	95.90	97.23

**FIGURE 5:**
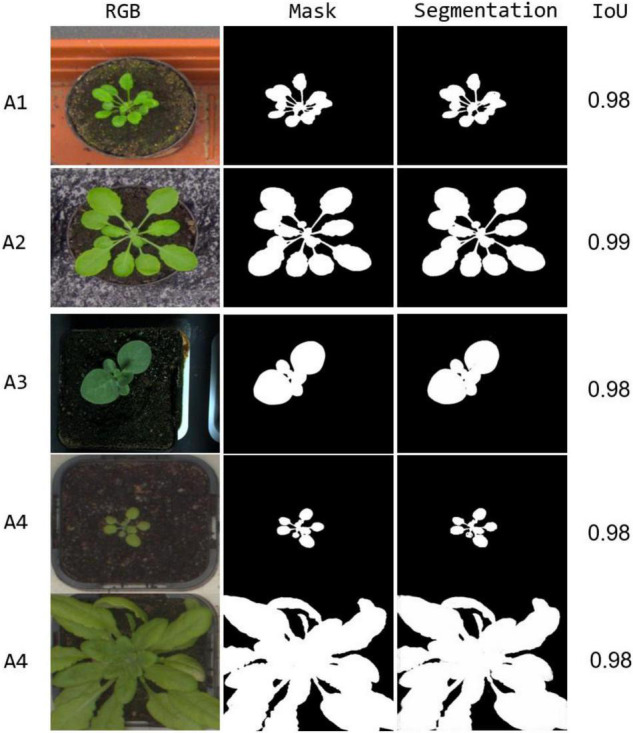
Segmentation result for each sub-dataset, with the corresponding IoU provided at the right.

During the training for segmentation, the sigmoid function produces outputs in the range [0 … 1]. While calculating the loss, greater weight is assigned for the boundary pixels. The weight map is then calculated using


(14) $$w\,\left(x\right)=w_{c}\,\left(x\right)+w_{0}\cdot \text{exp}\,\left(-\frac{{\left(d_{1}\,\left(\text{x}\;+\right)\,d_{2}\,\left(x\right)\right)}^{2}}{2\,\sigma^{2}}\right)$$

where *w*_*c*_(x) is the category weight based on the frequency of occurrence of each category in the training dataset; *d*_1_(x) represents the distance between the object pixel and the nearest boundary. *d*_2_(x) represents the same distance for the second nearest boundary. In our work, we set the threshold σ to 0.5 to obtain the segmentation weight map. The segmentation results using our method on different sub-datasets are shown in Figure 5. Our model generates the segmentation results that are almost coincident visually with the ground-truth mask for each sub-dataset. For A3 sub-dataset which only contains 27 tobacco images with small leaf area, our method still shows a stable segmentation result. The results show our method effectively addresses segmentation under various scenes, i.e., with occlusions, small leaf area, and large leaf area, demonstrating good robustness.

**FIGURE 6:**
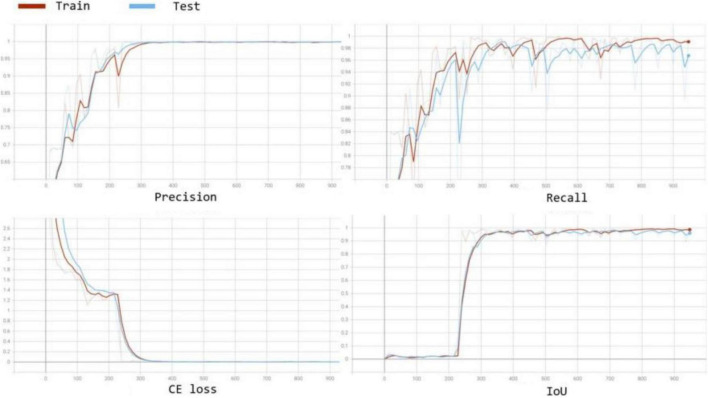
Convergence curves for accuracy, loss, and IoU score on the validation set during the training process for comparison in terms of accuracy and convergence rate.

**FIGURE 7:**
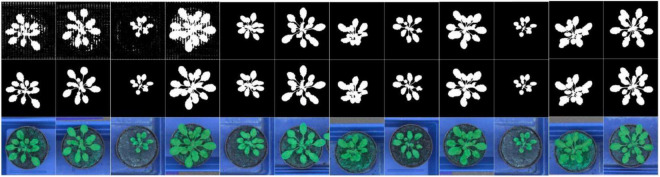
Visualization for the feature extraction process of our method, arranged by time series from the early to late epochs. The first to third line images respectively show the predicted images, ground truth images and transformed RGB images.

We also compared the convergence rate of different segmentation models. The curves of the precision, recall, training cross entropy (CE) loss, and IoU are shown in Figure 6. The figure shows that all four networks selecting VGG16 as the encoder for feature extraction achieve good IoU scores consistently. In addition, Figure 7 visualises the feature extraction process of our method using UNet++ with VGG from the early to late epochs. The process of feature extraction is smoother and faster to reach the convergence, which shows VGG can capture the meaningful representations for leaf images.

We compared the proposed segmentation model with the other state-of-the-art method that performed the experiment on plant (foreground) segmentation, i.e., SRGB (Aich and Stavness, 2017) using three metrics, i.e., Precision, Recall, and IoU and the results are shown in Table 3. Our method outperforms the SRGB method on two metrics, achieving the high performance on IoU. The results suggest that our approach is very effective for plant segmentation task in plant phenotyping.

**TABLE 3:** Segmentation results on each sub-dataset and their combination using different basic architectures.

	SRGB					Ours				
	All	A1	A2	A3	A4	All	A1	A2	A3	A4
Precision	0.92	0.98	0.94	0.80	0.96	0.99	0.99	0.99	0.99	0.99
Recall	0.97	0.99	0.99	0.94	0.98	0.99	0.98	0.99	0.99	0.99
IoU	–	–	–	–	–	0.98	0.98	0.99	0.98	0.98

#### Leaf Count Evaluations

In the second experiment, we evaluated the effectiveness of the proposed leaf counting method using segmented binary mask (referred as RGB+SBM). During the experiment, the number of input channels must be consistent with the input size of the backbone models, i.e., 3 channels. In this way, when a binary image with single channel is fed into the model, the values of the single channel are extended to three channels by duplication, forming an image with three channels. The resulting three-channel images are mixed with the RGB image samples to increase the number of training samples, facilitating the stability of leaf counting. To validate the effectiveness of our counting model for leaf counting, we adopted different backbones for our leaf counting task, e.g., MobileNet, VGGNet, InceptionNet, and ResNet, and report the results in Table 4. Moreover, to further explore the potential benefit of the auxiliary binary mask, we conducted an ablation experiment on with/without using the binary channel, and the result is also shown in Table 4. In Table 4, RGB denotes the method without using the binary mask, while RGB+SBM denotes that our method using the auxiliary binary mask. It is observed from the table that the count model using the ResNet50 backbone performs the best among the backbones. The binary mask increases the count performance in all metrics, where the MSE drops from 0.89 to 0.04, DiC from 0.02 to 0.01, and ADiC from 0.60 to 0.36. These results validate our assumption that binary mask improves the accuracy and robustness for the leaf count model, due to its capability to deal with background interferences.

**TABLE 4:** Counting results using different backbones with or without the auxiliary binary mask on CVPPP 2017 dataset (Bold values denote the best performance).

Metric	DiC	ADiC	MSE
**Mobilenet**			
RGB	–0.30	0.66	0.98
RGB+SBM	0.13	0.46	0.64
**InceptionNet**			
Rgb	0.29	0.61	1.20
RGB+SBM	0.20	0.43	0.54
**VGGNet**			
RGB	0.20	0.79	1.44
RGB+SBM	–0.12	0.37	0.44
**Resnet50**			
RGB	–0.12	0.60	0.89
RGB+SBM	**0.11**	**0.36**	0.42

*For DiC, ADiC, and MSE, a lower value is better.*

**FIGURE 8:**
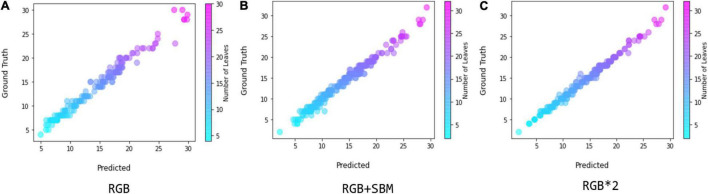
Comparison between the coefficient of determination in the implementation of scatter graphics, where **(A)** denotes using only RGB image, **(B)** denotes using the mixture of RGB and segmented binary mask, and **(C)** denotes using the double RGB images by simple copy.

We used the scatter diagram to visually illustrate the correlation between the estimated leaf numbers and their ground truth, and the results are shown in Figure 8, which is also for the evaluation of our regression model. The higher overlap between the scatter plots of estimation and the ground truth indicates a better agreement. Figure 8 shows that the binary mask significantly enhances the agreement between the ground truth and the estimation, as the error distribution in leaf count is constantly confined within smaller region. If directly doubling the number of the input samples by simple copy, referred as RGB *2, we find that the performance is almost the same as with the mixture of RGB and binary mask images. In the experiments, the time cost using double RGB images is higher than using the combination of RGB and binary mask images. Thus, we conclude that using the auxiliary binary mask to guide the leaf counting is a simple but effective way for improving the performance of counting.

In addition, we reported the quantitative comparison of our leaf counting method with state-of-the-art methods i.e., GLC (Giuffrida et al., 2015), IPK (Pape and Klukas, 2015), Nottingham (Scharr et al., 2016), MSU (Scharr et al., 2016), and Wageningen (Scharr et al., 2016), as shown in Table 5. For fair comparison, we used A1, A2, A3 from testing set for testing the counting performance. Overall, the proposed leaf counting model using segmented binary mask achieves the best performance with lower values in the metrics of DiC, ADiC, and MSE. This shows the proposed counting model estimates the number of leaves with adequate accuracy and stability.

**TABLE 5:** Comparative evaluation of the proposed counting model with state-of-the-art methods.

	DiC	ADiC	MSE
IPK	–1.9 (2.7)	2.4 (2.1)	–
GLC	–0.51 (2.02)	1.43 (1.51)	4.31
Nottingham	–2.4 (2.8)	2.9 (2.3)	–
MSU	–2.3(1.8)	2.4 (1.7)	–
Wageningen	1.5 (4.4)	2.5 (3.9)	–
Proposed RGB+SBM	0.11 (0.98)–	0.36 (0.93)	0.42

## Conclusion

In this study, we focus on dealing with two fundamental tasks in plant phenotyping, i.e., plant segmentation and leaf counting, and propose a two-stream deep learning framework for automatic segmenting and counting leaves with various size and shape from two-dimensional plant images. In the first stream, a multi-scale segmentation model using spatial pyramid is developed to extract the whole plant in different size and shape, where the fine-grained details of leaves are captured using deep feature extractor. In the second stream, a regression counting model is proposed to estimate the number of leaves without any pre-detection, where the auxiliary binary mask is introduced to enhance the counting performance by effectively alleviating the influence of complex background. Extensive experiments on a publicly available plant phenotyping dataset show that the proposed framework achieves a promising performance both in the task of plant segmentation and leaf counting, providing a reference for the automatic analysis of plant. Future work will focus in increasing the robustness of the tasks of segmentation and the counting to deal with varying environments.

## Data Availability Statement

Publicly available datasets were analyzed in this study. This data can be found here: https://www.plant-phenotyping.org/CVPPP2017.

## Author Contributions

XF contributed to writing the draft and designing the ideas. RZ contributed to conducting experiments. TT contributed to editing the draft. SD and QY contributed to algorithm supervision. All authors contributed to the article and approved the submitted version.

## Conflict of Interest

The authors declare that the research was conducted in the absence of any commercial or financial relationships that could be construed as a potential conflict of interest.

## Publisher’s Note

All claims expressed in this article are solely those of the authors and do not necessarily represent those of their affiliated organizations, or those of the publisher, the editors and the reviewers. Any product that may be evaluated in this article, or claim that may be made by its manufacturer, is not guaranteed or endorsed by the publisher.
